# Developing a carpet cloak operating for a wide range of incident angles using a deep neural network and PSO algorithm

**DOI:** 10.1038/s41598-023-27458-x

**Published:** 2023-01-12

**Authors:** Amirhossein Fallah, Ahmad Kalhor, Leila Yousefi

**Affiliations:** School of Electrical and Computer Engineering, College of Engineering, University of Tehran, Tehran, Iran

**Keywords:** Electrical and electronic engineering, Applied physics

## Abstract

Designing invisibility cloaks has always been one of the most fascinating fields of research; in this regard, metasurface-based carpet cloaks have drawn researchers' attention due to their inherent tenuousness, resulting in a lower loss and easier fabrication. However, their performances are dependent on the incident angle of the coming wave; as a result, designing a carpet cloak capable of rendering objects under it invisible for a wide range of angles requires advanced methods. In this paper, using the Particle Swarm Optimization (PSO) algorithm, along with a trained neural network, a metasurface-based carpet cloak is developed capable to operate for a wide range of incident angles. The deep neural network is trained and used in order to accelerate the process of calculation of reflection phases provided by different unit cell designs. The resultant carpet cloak is numerically analyzed, and its response is presented and discussed. Both near-field and far-field results show that the designed carpet cloak operates very well for all incident angles in the range of 0 to 65 degrees.

## Introduction

Concealing an object using invisibility cloaks has now turned from fiction into a scientific reality thanks to the pioneering work done by Pendry and Leonhardt^1^. After that, many researchers around the world were inspired to develop different cloaks making things invisible at different frequency regimes^1–22^. Initial cloaks were designed based on transformation optics consisting of a bulky material with both anisotropy and inhomogeneity, making its realization a difficult challenge^1–3^. To address this issue, other cloaking methods were proposed to make the designed cloaks realizable. One of these methods is carpet cloaking, a suitable way to hide objects on a ground plane, in which by tailoring the scattered waves from the designed cloak, the objects covered by the cloak appear as a flat ground plane^4–22^.

Carpet cloaks could be designed based on the quasi-conformal mapping method^4–9^; however, not only are these cloaks bulky in size, they generate a lateral shift in the scattered waves, resulting in the detection of the objects^23^. Another method is to use metasurfaces, ultrathin structures consisting of meta-atoms designed to introduce abrupt phase changes across its interface, tailoring the scattered electromagnetic wavefronts^10–22,24–33^. Metasurface-based carpet cloaks have several advantages over other cloaking methods, including lower loss, more convenient fabrication, lightweight, and ultrathin thickness^10–22^.

Although using metasurfaces to design a carpet cloak has the aforementioned advantages, it has intrinsic drawbacks; one of them is the dependence of the carpet cloak performance on the incident angle of the incoming electromagnetic waves; the maximum incident angle band reported has been 15°. To address this issue, an active, reconfigurable metasurface has been recently proposed^21^, in which a detector is used to acquire the information of the incoming wave including its angle of incidence. Then, the metasurface-based carpet cloak is adjusted using bias voltages to render the object invisible for that particular incident angle. Even though this active carpet cloak can work for different incident angles, there are still some drawbacks, such as complexity, delay (due to the detectors used to retrieve the information of the incident wave), and power consumption. Furthermore, it would be impossible to adjust the carpet cloak for two incoming waves with different incident angles simultaneously. Therefore, researchers are still encouraged to design a passive carpet cloak capable of rendering the covered objects invisible for a wide range of incident angles, although it would be arduous.

Deep neural networks are always considered for solving complicated problems which are difficult to solve by conventional, classic ways. In Electromagnetic problems, they have been used to model and analyze antennas^34–36^, imaging systems^37,38^, photonic devices^39,40^, and metasurfaces^21,41–46^.

In this work, a single-layer passive metasurface has been proposed, capable of rendering the objects under it invisible for a wide range of incident angles. The proposed metasurface consists of 15 unit cells, each designed using the Particle Swarm Optimization (PSO) algorithm^47^ in combination with a deep neural network, which is trained to solve Maxwell's equations much faster than a full-wave simulator. As a result, the optimum unit cells with reflection phases close to the one required by the carpet cloak have been found in a large data space. The reflection phase error of the unit cells achieved from the PSO algorithm is one-sixth of the best one existed in the initial training dataset, indicating the importance of using the PSO algorithm. Section 2 discusses the proposed structure; then, the whole designing process will be discussed in section 3. Finally, in section 4, the entire cloak is analyzed using a full-wave numerical simulator, and near-field and far-field results are presented. The results indicate good performance for all the incident angles in the range of 0° to 65°. As a result, the designed carpet cloak, which lacks the aforementioned drawbacks of the active carpet cloak due to its passive nature, is capable to operate well for a wide range of incident angles.

## The proposed structure and background theory

**Figure 1:**
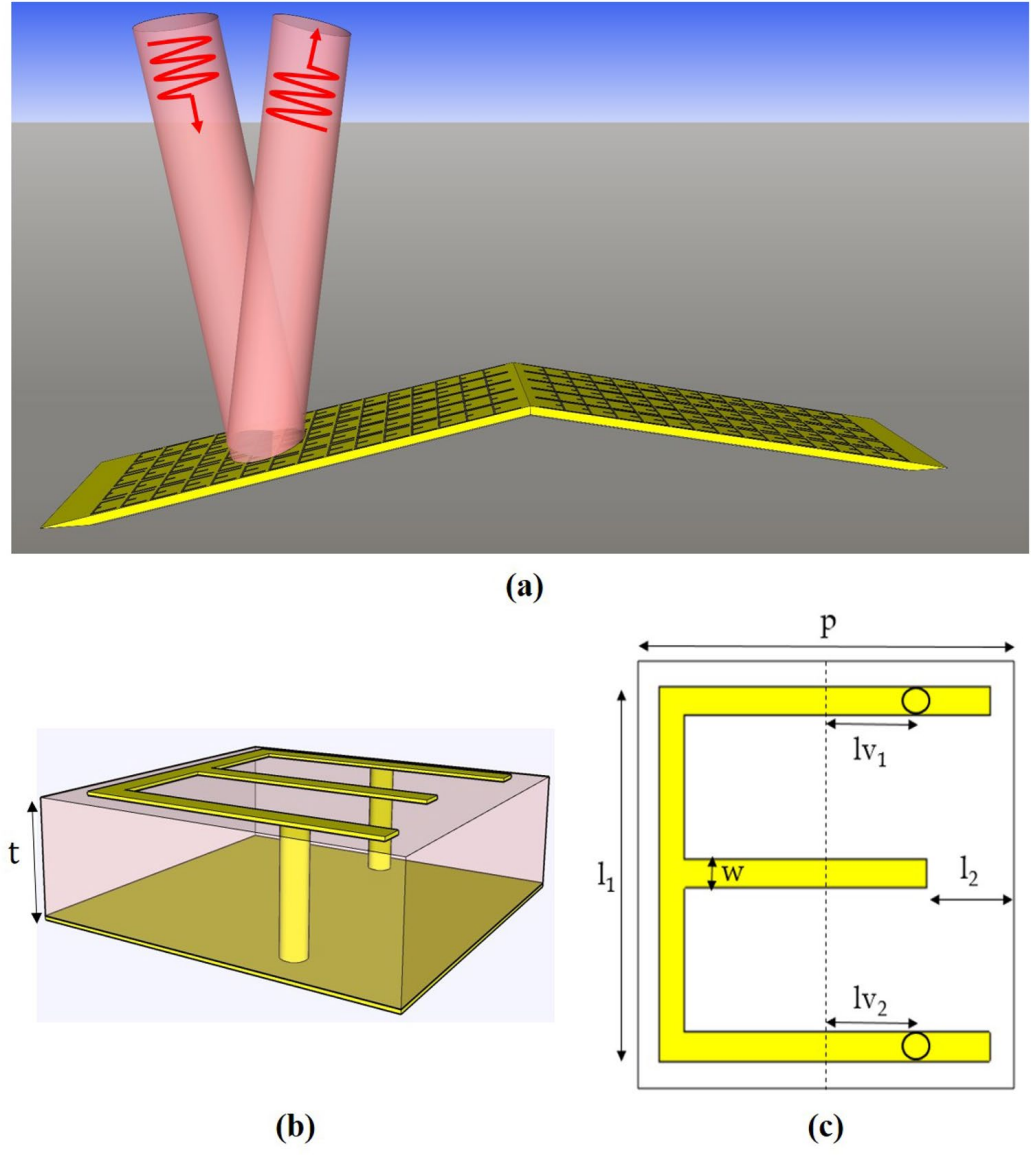
(**a**) Carpet invisibility cloak concealing objects under it consisting of 15 rows, each with a specific unit cell design. The structure of proposed unit cells' (**b**) perspective view, and (**c**) top view. The parameters *p*, *t*, and *w* remain constant, while the other parameters, *l*_*1*_, *l*_*2*_, *l*_*v1*_, and *l*_*v2*_, are tuned to generate different designs.

In this section, the proposed metasurface-based carpet cloak and the general structure of its unit cell are introduced. The proposed carpet cloak consisting of single-layered unit cells is shown in Fig. 1a**.** In order to tailor the scattered waves in such a way that they become similar to scattered waves from a flat ground with no object on it, each unit cell located at the height of *h* should provide the reflection phase of ^13^

1 $${\varphi }_{r}=\pi -2{k}_{0}hcos(\theta )$$

where *k*_*0*_ is the wave number in free space, and *θ* is the incident angle. Therefore, unit cells placed at different heights should have different designs to induce different reflection phases. Furthermore, according to Eq. (1)**,** the inquired reflection phase is a function of the incident angle. Thus, each unit cell should simultaneously provide suitable different reflection phases for all incident angles. Therefore, achieving such a competent design requires suitable unit cells and advanced designing tools. To achieve the mentioned goals, the structure shown in Fig. 1b,c is chosen as the unit cell and a deep neural network is trained and used to achieve an optimum structure that simultaneously provides all inquired reflection phases**.** As shown in Fig. 1b,c, the proposed unit cell includes an E-shaped copper planar structure with a copper trace width of *w* = *0.4 mm*, connected to its ground by two copper vias. The substrate is Rogers RO4003C with a thickness of *t* = *1.6 mm*, a relative permittivity of *ε*_*r*_ = *3.5*, and *tanδ* = *0.0027*; moreover, the unit cell has a periodicity of *p* = *6 mm.*

As shown in Fig. 1c, we have 7 design parameters for our unit cell. Four of these parameters (*l*_*1*_, *l*_*2*_, *l*_*v1*_, *l*_*v2*_) are considered variable and will be used in the optimization process to achieve our goal, while other 3 parameters (*p, w, t*) are remained constant. The periodicity of unit cells, *p,* by its definition, should be considered constant for all unit cells. The other two parameters are considered to be constant due to the limitations dictated by the fabrication process. We have designed the proposed carpet cloak in such a way that it could be fabricated by standard Printed Circuit Board (PCB) technology. To make this possible, *t*, which is the thickness of the substrate should be constant and equal to the thickness of available substrates. The diameter of vias, *w,* is also selected to be fixed to avoid expensive fabrication, since having vias with different diameters dramatically increases the fabrication cost.

When the incidence is oblique with TM polarization, a longitudinal electric field component appears, becoming more dominant as the incident angle increases. The unit cell should be able to exploit the longitudinal electric field differences between incident waves with different incident angles. That makes the electromagnetic response of the unit cells to incident waves with different incident angles, more diverse, resulting in the generation of disparate reflection phases for different incident angles.

To address this issue, as shown in Fig. 1b, longitudinal vias have been used in the structure of the proposed unit cell. Since the longitudinal electric field component induces a longitudinal electric current on vias, any change in the incident angle deeply affects this induced current, changing the reflection phase. Therefore, the proposed unit cell is expected to generate different reflection phases as the incident angle varies.

## Design process

This section describes the process of finding the best unit cells for the metasurface-based carpet cloak. The goal is to find 15 unit cell designs that induce the proper reflection phase (according to Eq. (1)) for 5 different incident angles, 10°, 25°, 40°, 55°, and 70°. Since in previously published works, a beamwidth of 15° has been reported for metasurface-based carpet cloaks, the designed cloak is expected to operate well for all incident angles in the range of 0° to 70°.

In the design process, the PSO algorithm is used to search through the design data space, which includes 4 design parameters of *l*_*1*_, *l*_*2*_, *l*_*v1*_, and *l*_*v2*_, to find the optimum unit cells. When searching for the optimum design, the reflection phase and magnitude of the unit cells should be calculated very fast. Full-wave simulations are not fast enough to be practically used to search for the optimum design. Therefore, here in this work, a deep neural network is designed and trained to get 4 designing parameters as input data, and generate the reflection phases and magnitude for the mentioned 5 incident angles, as the output data.

The whole process could be divided into 3 steps: generating a training dataset to train the neural network, designing and training a deep neural network using training data set, and performing the PSO algorithm 15 times to find the best designs for the unit cells while the trained neural network generates reflection phases and magnitudes within the algorithm (See Fig. S1 in the supplementary materials.)

### Generating training dataset

In order to train a deep neural network, a training dataset, including 4-feature input data, demonstrating 4 tunable parameters of the unit cells, and their corresponding output data, which are reflection phase and magnitude of input data, is required. First, 3240 different designs of the unit cell are considered as input data; which are achieved by choosing different values for *l*_*1*_, *l*_*2*_, *l*_*v1*_, and *l*_*v2*_ (see Table S1 in the supplementary materials for more details.) Afterward, these input data are numerically simulated using the CST Microwave Studio, a commercial full-wave solver that numerically solves Maxwell's equations, to achieve their corresponding output data, which are reflection phases and magnitudes for incident angles of 0°, 15°, 30°, 45°, and 60° (since the cloak's tilt angle is 10°, each incident angle with respect to the metasurface would be 10° less than the incident angle with respect to the ground). Further details about the simulation setup can be found in the methods section.

### Design and train of a deep neural network

After generating the training dataset, a deep neural network is designed to solve the forward problem, mapping unit cell designs to reflection phases and amplitudes. Before training the neural network, a pre-processing of the training data is required. In this pre-processing, input data and reflection magnitudes are scaled to the [-1,1] band. Furthermore, since the reflection phases have a periodic nature, which can confuse the neural network, instead of using reflection phases as outputs in the neural network, their sine and cosine functions are considered as output data. As a result, output data are 15-feature data, corresponding to the sine and cosine of reflection phases and reflection magnitude for the 5 mentioned incident angles.

**Figure 2:**
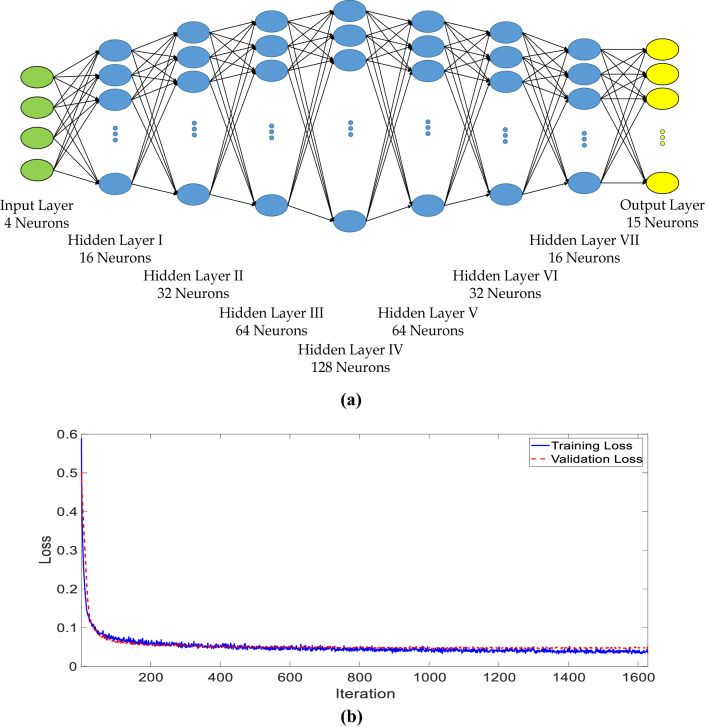
(**a**) The topology of the designed deep neural network including 7 hidden layers with decoder-encoder shape. (**b**) Training loss and validation loss through the training process.

The topology of the designed neural network is shown in Fig. 2a. It is realized that due to the complexity of the problem, high-feature basis functions are required to make the learning process feasible. Therefore, as shown in Fig. 2a, the topology of the designed network is analogous to a decoder-encoder topology. As a result, the 4-feature input data would be mapped to 128-feature data in the middle layer, representing these high-feature basis functions; then, they are mapped into 15-feature output data.

The activation functions of the hidden layers and the output layer are *Relu* functions^48^ and *tanh* function^48^, respectively. Mean Squared Error (MSE)^48^ is used as the loss function, and the optimizer is *Adam*^49^. In order to accelerate the training process, batch normalization layers^50^ are applied before each hidden layer. Furthermore, instead of setting a maximum number of iterations in the training process, an early stopping condition^51^ has been applied for the validation loss. 80% of the generated dataset (see “Details on the Training Dataset” part in the supplementary information, for more details) is used for training the network and 20% of it is used as the validation dataset. The validation dataset is chosen randomly, and take no part in the training process. As a result, the validation loss could be considered as an important factor to accurately evaluate the performance of the trained neural network. The training and validation loss functions throughout the training process are shown in Fig. 2b. As shown in this figure, after 1628 epochs, the training process stops. Although the loss functions have been reduced significantly in the first 50 epochs, the process continued due to small reductions in the validation loss. It is worth mentioning that other topologies and number of hidden layers have been also considered and investigated before finalizing the network. However, our study shows that the training and the validation loss do not attenuate well in the case of using lower number of hidden layers, and for the case of higher number of hidden layers, higher validation loss is achieved.

**Figure 3:**
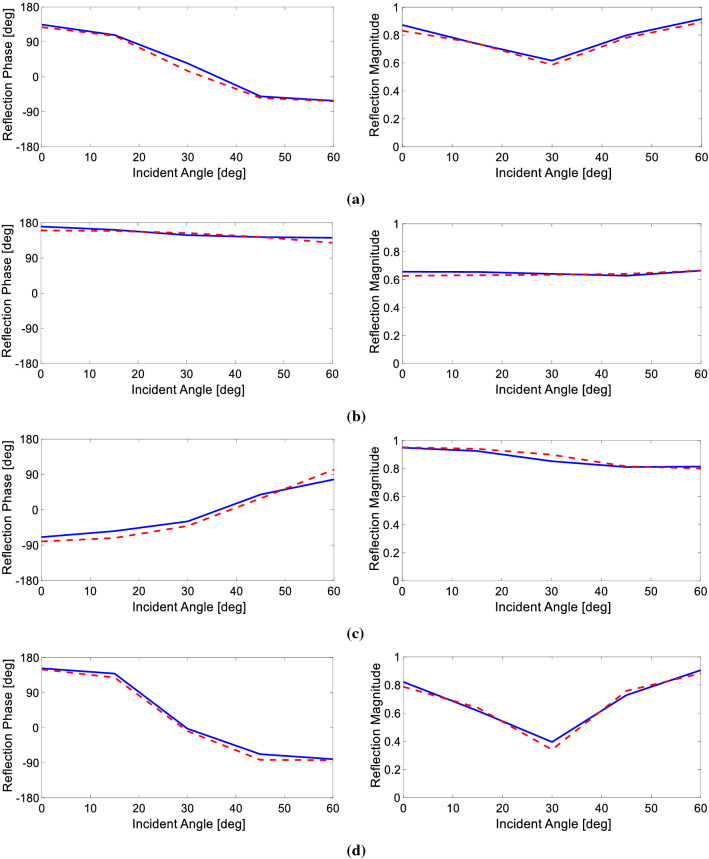
Reflection phases and magnitudes of 4 random validation data with design parameters of (**a**) *l*_*1*_ = *5.6 mm*, *l*_*2*_ = *4.5 mm*, *l*_*v1*_ = *-1.5 mm*, *l*_*v2*_ = *0.0 mm*, (**b**) *l*_*1*_ = *5.6 mm*, *l*_*2*_ = *0.0 mm*, *l*_*v1*_ = *-0.3 mm*, *l*_*v2*_ = *-1.2 mm* $,$ (**c**) *l*_*1*_ = *5.3 mm*, *l*_*2*_ = *2.0 mm*, *l*_*v1*_ = *-1.2 mm*, *l*_*v2*_ = *-1.2 mm* $,$ and (**d**) *l*_*1*_ = *5.6 mm*, *l*_*2*_ = *5.0 mm*, *l*_*v1*_ = *-1.2 mm*, *l*_*v2*_ = *1.5 mm*, for different incident angles, achieved from numerical simulations (blue diagrams) and the trained neural network (dashed red diagrams).

For further evaluations, 4 random data from the validation data have been selected, and the response achieved from the designed neural network for these 4 structures is compared with the response achieved from accurate full-wave numerical simulation. This comparison is shown in Fig. 3. As shown in this figure, the reflection phases and magnitudes achieved from the neural network are similar to the accurate responses, indicating that the neural network could be relied on to generate accurate reflection phases and magnitudes for the PSO algorithm.

### Designing the carpet cloak using PSO algorithm

After training a deep neural network capable of generating reliable reflection phases and magnitudes for new designs, in this section, 15 suitable unit cells have been achieved using the PSO algorithm. The PSO algorithm is one of the evolutionary algorithms^52^, capable of finding optimums in a large data space, used in many engineering designs^53^. The algorithm procedure is illustrated in Fig. S2, which can be described as finding the optimum point in the design data space, where each point is a 4-feature vector indicating the values of the parameters *l*_*1*_, *l*_*2*_, *l*_*v1*_, and *l*_*v2*_.

First, a target is determined, which includes required reflection phases based on equation (1), and reflection magnitudes of 0 [dB] for all the 5 mentioned incident angles (see Table S2 of the Supplementary Materials). Afterward, a criterion for each point in the design data space should be defined based on how their corresponding reflection phases and magnitudes for different incident angles are close to the desired values. For this purpose, a score function is defined as

2 $$ Score = 100 - 200\frac{{\left\| {{\varvec{F}}_{P} \left( {{\varvec{X}}_{i} } \right) - {\varvec{Y}}_{Target} } \right\|}}{10} - 10\frac{{\left\| {{\varvec{F}}_{M} \left( {{\varvec{X}}_{i} } \right)} \right\|}}{5} $$

where ${{\varvec{X}}}_{i}$ represents i-th particle 4-feature vector consisting of values of the parameters *l*_*1*_, *l*_*2*_, *l*_*v1*_, and *l*_*v2*_, ${{\varvec{Y}}}_{Target}$ represents a 10-feature vector consisting of the sine and cosine of the target reflection phases, ${{\varvec{F}}}_{P}\left({{\varvec{X}}}_{i}\right)$ represents the 10-feature vector consisting of the sine and cosine of the reflection phases achieved from the neural network for the design corresponding to ${{\varvec{X}}}_{i}$, and ${{\varvec{F}}}_{M}\left({{\varvec{X}}}_{i}\right)$ represents the 5-feature vector consisting of reflection magnitudes achieved from the neural network for the design corresponding to ${{\varvec{X}}}_{i}$. Both ${{\varvec{F}}}_{P}\left({{\varvec{X}}}_{i}\right)$ and ${{\varvec{F}}}_{M}\left({{\varvec{X}}}_{i}\right)$ are calculated using the designed neural network in less than seconds, indicating its significant contribution in calculating the unit cell design score and improving the process speed. The second term in Eq. (2) is responsible for finding a design whose reflection phases are closest to the desired value, while the third term is responsible for its reflection magnitudes; thus, the coefficients (200 and 10) are assigned based on the level of importance of each part.

After determining the score function, 100 particles are distributed in the design space with random initial positions and velocities; these particles are able to communicate with each other, reporting their best-experienced positions with the highest score, called "local optimums." Moreover, the best local optimum among these particles with the highest score is called the "global optimum." Then, in each iteration, their positions are updated as

3 $${{\varvec{X}}}_{i,t}={{\varvec{X}}}_{i,t-1}+{{\varvec{V}}}_{i,t}$$

where ${{\varvec{X}}}_{i,t}$ , ${{\varvec{V}}}_{i,t}$ represent the i-th particle position, and speed at the time of *t*, respectively. The velocity of particles are updated as

4 $${{\varvec{V}}}_{i,t}=w.{{\varvec{V}}}_{i,t-1}+{c}_{1}.rand.\left({{\varvec{P}}}_{i,t-1}-{{\varvec{X}}}_{i,t-1}\right)+{c}_{2}.rand.\left({{\varvec{P}}}_{g,t-1}-{{\varvec{X}}}_{i,t-1}\right)$$

where ${{\varvec{P}}}_{i,t-1}$ represents i-th particle local optimum at the time of $t-1$, ${{\varvec{P}}}_{g,t-1}$ represents the global optimum at the time of $t-1$*,* and *rand* is a random number between 0 to 1, giving stochastic nature to the algorithm. The hyperparameters *c*_*1*_ and *c*_*2*_ are assigned as 0.1 and 0.2, respectively. These values are selected through a trial and error process. After 40 iterations, the global optimum is considered as the best unit cell design with reflection phases and magnitudes closest to the desired values.

After running the algorithm 15 times, the optimum unit cells for 15 different heights, each requiring different reflection phases based on equation (1), are achieved. The dimensions of the finalized unit cells are shown in Table S3 of the Supplementary Materials. Further investigation of the PSO algorithm could be found in the Supplementary Materials, as Fig. S3 shows the score changes of particles selected as the global optimum for these 15 runs. Moreover, for further evaluation, the optimum designs achieved from the PSO algorithm are numerically analyzed using two different solvers of CST Microwave Studio and Ansys HFSS software, and their reflection phases and magnitudes for different incident angles are calculated and shown in Tables S4 and S5 of the Supplementary Materials. Subsequently, simulation results are used to calculate the real scores and phase error of unit cells using equation (5):

5 $$ {\varvec{E}}_{i} = \frac{{\left\| {\phi_{sim} - \phi_{req} } \right\|}}{5} $$

where ${{\varvec{E}}}_{i}$ is the error of the i-th unit cell, ${{\varvec{\phi}}}_{sim}$ is a 5-feature vector consisting of reflection phases for the 5 mentioned incident angles achieved from the simulations, and ${{\varvec{\phi}}}_{req}$ is a 5-feature vector consisting of required reflection phases for the 5 mentioned incident angles. The unit cells' real scores and reflection phase errors are shown in Table 1.

**Table 1:** Calculated real scores and phase errors for finalized unit cells.

Unit cell number	1	2	3	4	5	6	7	8	9	10	11	12	13	14	15
Real scores	97.9	97.2	94.9	94.9	93.3	93.4	90.2	91.7	90.9	91.4	92.4	92.1	88.8	86.7	81.2
Phase error [deg]	1.09	1.40	2.49	2.54	3.38	3.36	5.09	4.13	4.59	4.39	3.55	3.64	4.77	6.40	9.63

As shown in Table 1, the unit cells have low reflection phase errors and high scores, and a reflection phase error average of 4.03° is achieved. It is worth mentioning that for the sake of comparison, another carpet cloak using the training dataset and without using the deep neural network and the PSO algorithm is designed. For that design, a reflection phase error average of 24.85°, 6 times higher than the proposed design, is achieved (Further Details in the Supplementary Materials). This comparison indicates the importance of the proposed design procedure.

## Results and discussion

**Figure 4:**
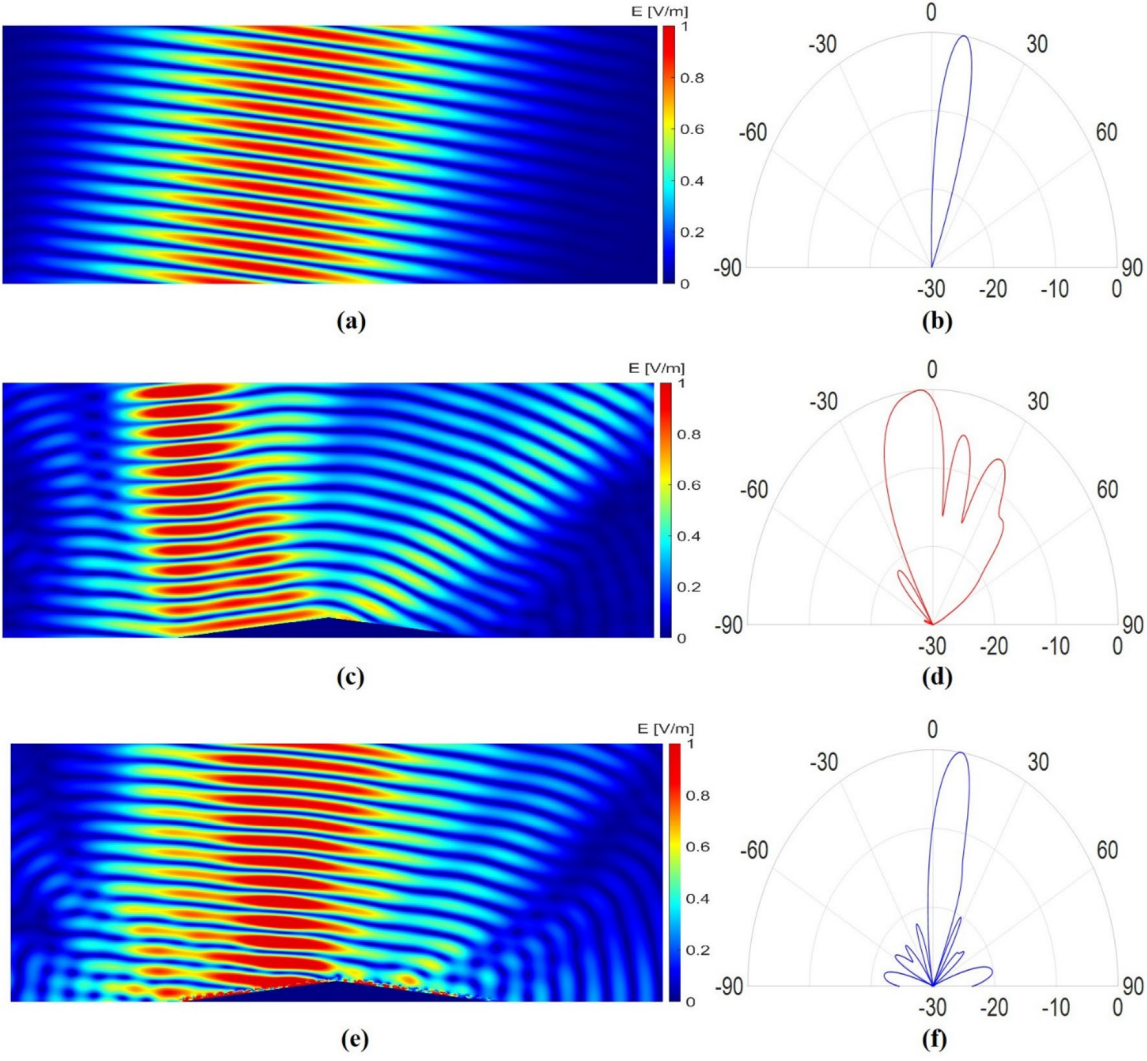
Simulation results for Gaussian wave incidence with the incident angle of 10°. (**a, c, e**) Near field reflected electric field distribution for a metallic ground plane, bare bump, and cloaked bump, respectively. (**b, d, f**) The normalized intensity of the scattered field (dB) for a metallic ground plane, bare bump, and cloaked bump, respectively.

In this section, the performance of the optimized cloak is evaluated by full-wave numerical simulations performed in Ansys HFSS software. In the simulations, a Gaussian beam with a waist radius of 90 mm is considered as the incident wave with different incident angles to analyze both near-field and far-field results. Figure 4 shows the scattered electric field distribution and the normalized intensity of the scattered wave for the incident angle of 10°. As shown in this figure, when the bare bump is exposed to the radiations (Fig. 4c,d), the direction of the reflected beam is completely deviated compared to a situation in which the bump is absent and there is only a metallic ground plane (Fig. 4a,b); which makes the bump detectable; however, when the bump is covered with the designed carpet cloak (Fig. 4e,f), the scattered beam is completely similar to the scattered beam from a metallic ground plane, making the bump and objects under it invisible.

**Figure 5:**
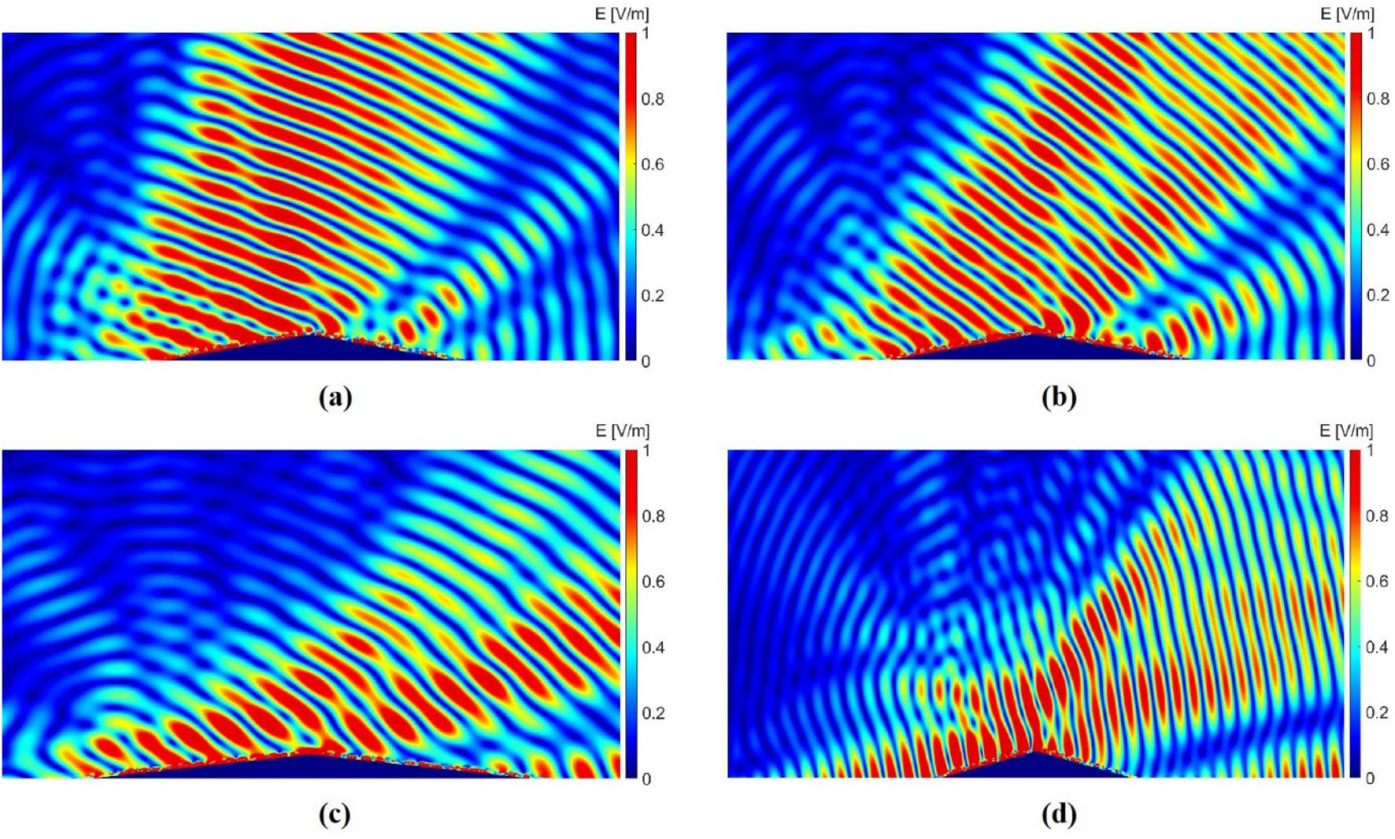
Reflected electric field distributions for the cloaked bump under Gaussian wave incidence with incident angles of (**a**) 25°, (**b**) 40°, (**c**) 55°, and (**d**) 70°.

**Figure 6:**
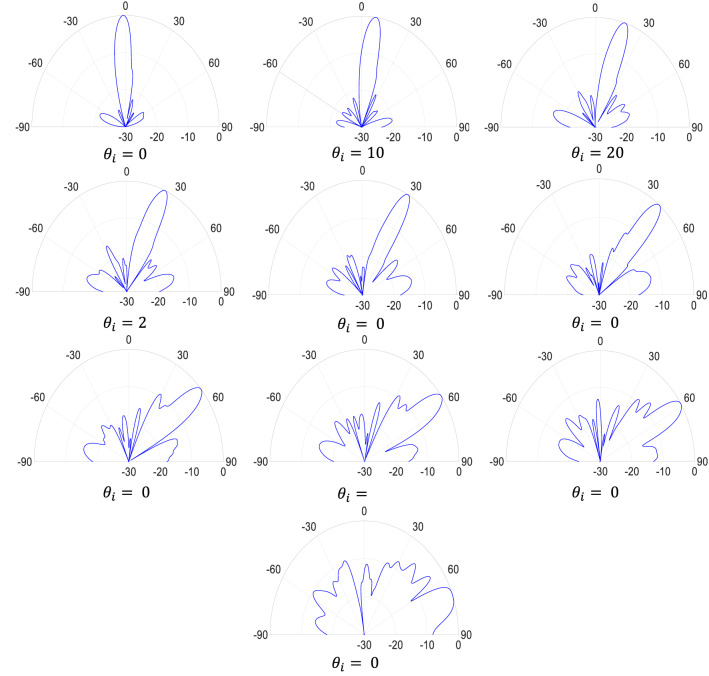
The normalized intensity of the scattered field (dB) for the cloaked bump under Gaussian wave incidence with incident angles in the range of 0 to 70 degrees.

Further simulations are performed to investigate the performance of the designed cloak for other incident angles. Figure 5 shows the near-field results for the other 4 main incident angles when the bump is covered by the designed carpet cloaks. As shown in Fig. 5, the scattered beam is in the same direction as it is reflected from the ground; nevertheless, the far-field results could be more helpful in evaluating the cloak performance. As mentioned in the design process section, the cloak is designed for 5 main incident angles, hoping that it would have a good performance for the other angles in between. Therefore, the cloak is simulated for incident angles in the range of 0 to 70 degrees. Figure 6 shows the normalized intensity of the scattered wave in the far-field region for the main incident angles and incident angles from 0 to 70 degrees with 10-degrees steps. As shown in Fig. 6, the direction of the scattered main beam is in the same direction as it is reflected from the ground, except for the 70-degree case, which its result is not as acceptable as the others. To demonstrate the performance of the cloak for all the incident angles, a motion picture could also be found in the supplementary materials, showing the far-field results with 5-degrees steps.

For further evaluations, here numerical criterion called Reduced RCS^13^ is used:

6 $${Reduced\, RCS}_{Cloaked/ Bare\, Bump}\left(\theta \right)=\frac{{\left|{{\varvec{E}}}_{Cloaked/ Bare\, Bump}(\theta )-{{\varvec{E}}}_{Ground}(\theta )\right|}^{2}}{\mathit{max}\left({\left|{{\varvec{E}}}_{Cloaked/ Bare\, Bump}\left(\theta \right)-{{\varvec{E}}}_{Ground}\left(\theta \right)\right|}^{2}\right)}$$

where ${{\varvec{E}}}_{Cloak}$, ${{\varvec{E}}}_{Bare Bump}$, and ${{\varvec{E}}}_{Ground}$ are the electric field scattered to the far-field region from the cloaked bump, bare bump, and the ground, respectively. Reduced RCS is a function of the incident angle *θ*, indicating that in the far-field region, at each *θ*, how close the scattered electric field is to the electric field scattered from the ground. Therefore, for the cloaking structures, less Reduced RCS at each *θ* infers better invisibility.

After calculating Reduced RCS, another numerical criterion could be defined asyy^13^

7 $$Reduced\, Total\, RCS= \frac{{\int }_{0}^{2\pi }{Reduced\, RCS}_{Cloaked}\left(\theta \right)d\theta }{{\int }_{0}^{2\pi }{Reduced\, RCS}_{Bare\, Bump}\left(\theta \right)d\theta }$$

that is the ratio of integrated Reduced RCS along *θ* values for the cloaked bump to the bare bump, indicating how small is the integrated Reduced RCS of the cloaked bump compared to the bare bump. Therefore, Reduced Total RCS is an appropriate numerical criterion showing the performance of the carpet cloak for each incident angle.

**Figure 7:**
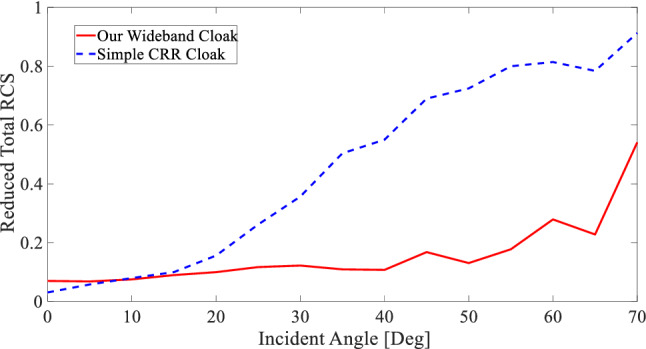
Reduced Total RCS for the proposed cloak (solid red diagram) is compared with that of a simple cloak designed using CRR unit cells (dash blue diagram).

For the sake of comparison, a simple carpet cloak is designed that operates for only normal incidents, using CRR unit cells (Further details in Supplementary Materials), and this criterion is also calculated for this cloak, for different incident angles. Figure 7 shows the Reduced Total RCS for both the proposed cloak and the mentioned simple cloak. It can be inferred from Fig. 7 that the proposed carpet cloak has much better performance in higher incident angles than a simple carpet cloak designed only to render the objects under it invisible for only one incident angle. Nonetheless, the value of Reduced Total RCS for the incident angle of 70° is higher than 0.5, which is not ideal for a carpet cloak. Therefore, it can stated that the carpet cloak is capable of rendering objects under it invisible for a wide range of incident angles, 0 to 65 degrees.

## Conclusion

In conclusion, a passive carpet cloak capable of operating over a wide range of incident angles from 0° to 65° is designed. Due to the intrinsic complexity of the problem, the PSO algorithm is used to perform an intelligent search through the design data space to find suitable unit cells for the metasurface-based carpet cloak. In order to execute the PSO algorithm in a fast way, a deep neural network capable of generating reliable reflection phases and magnitudes for different unit cell designs was trained and utilized. As a result, the phase error average has been decreased by a factor of 6 compared to a designing procedure excluding the deep neural network and the PSO algorithm.

After designing the carpet cloak, a set of simulations were performed, in which a Gaussian incident wave with different incident angles has stroke the carpet cloak to evaluate its performance by investigating near-field and far-field results, which indicate the acceptable performance of the carpet cloak. Moreover, for further evaluations, a numerical criterion was defined; and the cloak performance was compared with a simple cloak, designed to operate only for an incident angle of 0°. The comparison infers that the designed cloak has a much better performance for a wide range of incident angles.

For further improvement, the design could be developed to induce required reflection phases not only for different incident angles but also for different frequencies, resulting in an ideal passive carpet cloak.

## Methods

In this paper, the reflection phases and magnitudes of different forms of unit cells are calculated using CST Microwave Studio software. In the simulations for unit cells, which are performed at the frequency of 10 GHz, Floquet ports are used for excitation, and periodic boundary condition has been applied in both tangential directions.

Then, Tensorflow machine-learning platform is used to design the neural network using the achieved training dataset. Finally, Ansys HFSS software is used to simulate the whole carpet cloak and numerically calculate the scattering waves and evaluate the cloak performance at the frequency of 10 GHz.

## Supplementary Information

Supplementary Video 1.

Supplementary Information 1.

## Data Availability

The datasets generated and/or analyzed during the current study are available from the corresponding author upon reasonable request.
