# Supplementary material for: Developing a carpet cloak operating for a wide range of incident angles using a deep neural network and PSO algorithm
Source: Sci Rep. 2023 Jan 12;13:670. doi: 10.1038/s41598-023-27458-x (PMC9837171; doi:10.1038/s41598-023-27458-x)
Supplement: Supplementary file 2 — Supplementary Information 1. [file 41598_2023_27458_MOESM2_ESM.docx]

**Supplementary Information:**

**Developing a Carpet Cloak Operating for a Wide Range of Incident Angles using a Deep Neural Network and PSO Algorithm**

**Amirhossein Fallah^1^, Ahmad Kalhor^1^, and Leila Yousefi^1,*^**

^1^School of Electrical and Computer Engineering, University of Tehran, Tehran, postcode, Iran
^*^ lyousefi@ut.ac.ir

# Illustration of the Design Procedure and the PSO Algorithm.

The details of the design procedure is shown in Fig. [S1](#fsn1), and Fig. [S2](#fsn2) illustrates the details of the PSO algorithm.


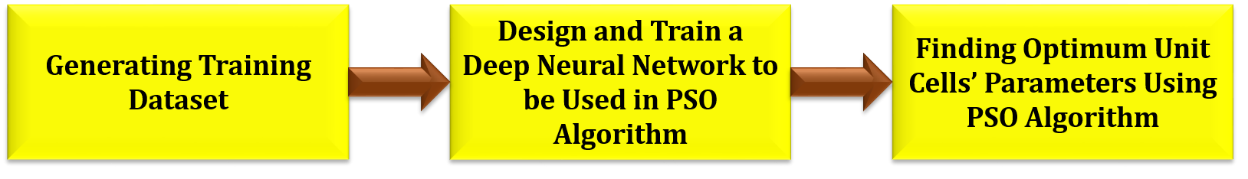


Figure S1. Overview of the design process.


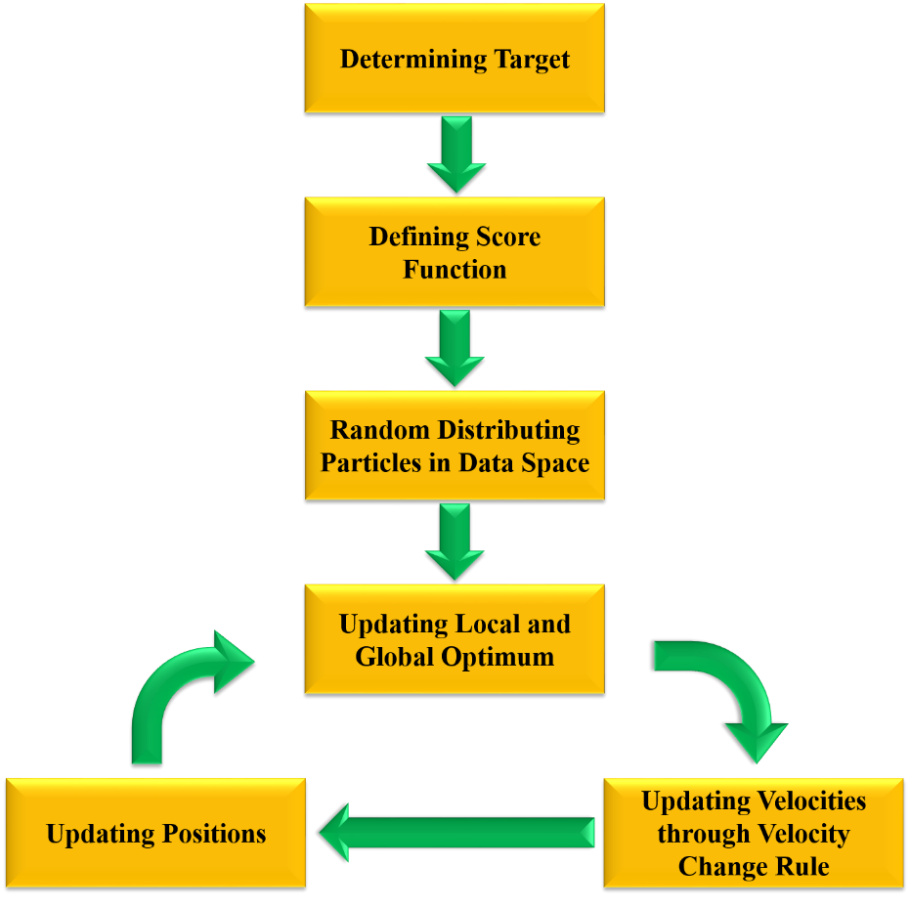


Figure S2. Illustration of PSO algorithm used along with the trained neural network to achieve the optimum design.

# Details on the Training Dataset:

The values of the 3 tunable parameters *l_1_*, *l_v1_*, and *l_v2_*, are chosen from the data shown in Table S1 to generate the training dataset. The other parameter, *l_2 ,_* starts from 0 mm and stops at its maximum possible value (*l_1_-w*) with 0.5 mm steps.

**Table** **S1.** Data used for design parameters in order to generate training dataset

| *l_1_* [mm] | 5.0 | | | 5.3 | | | 5.6 | | | 5.9 | | |
| --- | --- | --- | --- | --- | --- | --- | --- | --- | --- | --- | --- | --- |
| *l_v1_* [mm] | -1.5 | -1.2 | -1.0 | | -0.9 | -0.8 | -0.6 | -0.3 | 0.0 | | 0.9 | 1.5 |
| *l_v2_* [mm] | -1.5 | -1.2 | -1.0 | | -0.9 | -0.8 | -0.6 | -0.3 | 0.0 | | 0.9 | 1.5 |

# Procedure of PSO Algorithm and Evaluation of Achieved Results

The PSO algorithm is performed 15 times to achieve 15 unit cells inducing required reflection phases shown in Table [S2](#ts1).

**Table S2.** Required reflection phases for 15 unit cells [deg].

| Unit Cell Number/Incident Angle | 10° | 25° | 40° | 55° | 70° |
| --- | --- | --- | --- | --- | --- |
| 1 | 167.7 | 168.7 | 170.4 | 172.8 | 175.7 |
| 2 | 143.1 | 146.0 | 151.3 | 158.5 | 167.2 |
| 3 | 118.4 | 123.3 | 132.1 | 144.1 | 158.6 |
| 4 | 93.8 | 100.7 | 133.0 | 129.8 | 150.1 |
| 5 | 69.2 | 78.0 | 93.8 | 115.5 | 141.5 |
| 6 | 44.6 | 55.3 | 74.6 | 101.1 | 133.0 |
| 7 | 19.9 | 32.7 | 55.5 | 86.8 | 124.4 |
| 8 | -4.7 | 10.0 | 36.3 | 72.4 | 115.9 |
| 9 | -29.3 | -12.6 | 17.2 | 58.1 | 107.3 |
| 10 | -53.9 | -35.2 | -2.0 | 43.7 | 98.8 |
| 11 | -78.6 | -58.0 | -21.1 | 29.4 | 90.2 |
| 12 | -103.2 | -80.6 | -40.2 | 15.1 | 81.6 |
| 13 | -127.8 | -103.3 | -59.4 | 0.7 | 73.1 |
| 14 | -152.4 | -125.9 | -78.6 | -13.6 | 64.5 |
| 15 | -177.0 | -148.6 | -97.8 | -28.0 | 56.0 |

Figure [S3](#fs1) shows the score progress through performing the PSO Algorithm for particles selected as the global optimum for 15 different heights. The dimensions of the optimum unit cells for these 15 heights are listed in Table [S3](#ts2).

**Figure S3.** The score progress through applying the PSO algorithm shown for optimum particles locating at different heights.

After achieving the parameters of optimum unit cells, further evaluation is done by full-wave numerical simulation in both CST Microwave Studio and Ansys HFSS to numerically calculate reflection phases and magnitudes for each unit cell when illuminated at the aforementioned incident angles. The results are shown in Tables [S4](#ts3) and [S5](#ts4).

**Table S3.** Dimensions of the optimum unit cells.

| Unit Cell Number | l_1_ [mm] | l_2_ [mm] | l_v1_ [mm] | l_v2_ [mm] |
| --- | --- | --- | --- | --- |
| 1 | 5.00 | 3.86 | 0.35 | 0.94 |
| 2 | 5.15 | 2.95 | 0.13 | -0.37 |
| 3 | 5.30 | 2.94 | -0.35 | -0.70 |
| 4 | 5.06 | 2.01 | -0.43 | -0.69 |
| 5 | 5.17 | 2.26 | -0.68 | -0.74 |
| 6 | 5.09 | 1.89 | -0.78 | -0.78 |
| 7 | 5.36 | 2.41 | -0.63 | -0.82 |
| 8 | 5.37 | 2.41 | -0.88 | -0.77 |
| 9 | 5.30 | 2.17 | -0.98 | -0.89 |
| 10 | 5.60 | 3.00 | -0.80 | -0.80 |
| 11 | 5.49 | 2.69 | -0.90 | -0.93 |
| 12 | 5.60 | 3.01 | -0.90 | -0.90 |
| 13 | 5.90 | 3.00 | -0.60 | -1.50 |
| 14 | 5.88 | 4.19 | -0.79 | -0.78 |
| 15 | 5.89 | 4.27 | -0.80 | -0.83 |

**Table S4.** Numerically calculated reflection phases [deg] for the optimum unit cells.

| Unit Cell Number/Incident Angle | 10° | 25° | 40° | 55° | 70° |
| --- | --- | --- | --- | --- | --- |
| 1 | 169.7 | 170.2 | 172.0 | 175.2 | 179.6 |
| 2 | 147.8 | 149.8 | 154.2 | 160.4 | 166.4 |
| 3 | 109.8 | 116.2 | 131.4 | 147.8 | 162.6 |
| 4 | 90.4 | 96.2 | 110.2 | 126.4 | 139.6 |
| 5 | 63.2 | 73.6 | 98.8 | 127.0 | 150.0 |
| 6 | 50.2 | 59.8 | 84.4 | 112.4 | 135.6 |
| 7 | 25.8 | 35.4 | 59.6 | 85.4 | 100.2 |
| 8 | -9.0 | 2.2 | 34.2 | 72.2 | 97.4 |
| 9 | -29.4 | -19.0 | 12.0 | 54.5 | 86.2 |
| 10 | -53.2 | -46.1 | -5.1 | 44.9 | 78.1 |
| 11 | -67.2 | -57.4 | -23.8 | 32.4 | 77.2 |
| 12 | -88.0 | -78.4 | -44.3 | 24.0 | 81.8 |
| 13 | -112.4 | -104.8 | -73.0 | -0.6 | 61.2 |
| 14 | -131.8 | -125.6 | -98.6 | -13.2 | 78.6 |
| 15 | -139.8 | -134.2 | -112.4 | -34.2 | 77.8 |

**Table S5.** Numerically calculated reflection magnitude [dB] for the optimum unit cells.

| Unit Cell Number/Incident Angle | 10° | 25° | 40° | 55° | 70° |
| --- | --- | --- | --- | --- | --- |
| 1 | -0.05 | -0.05 | -0.06 | -0.08 | -0.11 |
| 2 | -0.09 | -0.10 | -0.13 | -0.16 | -0.23 |
| 3 | -0.28 | -0.28 | -0.30 | -0.32 | -0.40 |
| 4 | -0.17 | -0.18 | -0.22 | -0.28 | -0.38 |
| 5 | -0.25 | -0.27 | -0.32 | -0.37 | -0.48 |
| 6 | -0.20 | -0.22 | -0.29 | -0.37 | -0.50 |
| 7 | -0.42 | -0.43 | -0.45 | -0.44 | -0.46 |
| 8 | -0.42 | -0.46 | -0.52 | -0.55 | -0.57 |
| 9 | -0.36 | -0.40 | -0.52 | -0.62 | -0.68 |
| 10 | -0.34 | -0.39 | -0.50 | -0.53 | -0.46 |
| 11 | -0.35 | -0.41 | -0.61 | -0.80 | -0.79 |
| 12 | -0.32 | -0.40 | -0.67 | -1.00 | -0.89 |
| 13 | -0.69 | -0.82 | -1.35 | -1.97 | -1.34 |
| 14 | -0.24 | -0.30 | --0.63 | -1.60 | -1.10 |
| 15 | -0.19 | -0.24 | -0.52 | -1.69 | -1.31 |

# An Alternative Design without Using the PSO Algorithm

In order to illustrate the importance of the PSO algorithm, another carpet cloak is designed using unit cells found in the training dataset. In other words, the PSO algorithm is excluded from the design procedure and the best unit cells in the generated training dataset are considered as the final design. To select the required unit cells which are supposed to induce reflection phases close to those listed in Table [S2](#ts1), a loss function is defined for each design in the training dataset as follows:

$Error= 20\frac{\left\| \boldsymbol{y}_{simulated}-\boldsymbol{y}_{target} \right\|}{10}- \frac{\left\| \boldsymbol{y}_{M} \right\|}{5}$ (S1)

where $\boldsymbol{y}_{simulated}$ represents a 10-feature vector consisting of sines and cosines of reflection phases for the 5 aforementioned incident angles, calculated numerically using CST software, $\boldsymbol{y}_{target}$ represents a 10-feature vector consisting of sine and cosine of target reflection phases, and $\boldsymbol{y}_{M}$ represents a 5-feature vector consisting of reflection magnitudes calculated numerically using CST software. The loss function for all the designs in the training dataset is calculated and the design with the minimum loss function is selected. By repeating this procedure 15 times for 15 unit cells located at 15 different heights, the carpet cloak is designed. Afterward, unit cells are simulated using numerical full-wave analysis to calculate their reflection phases accurately. Then using equation (5), the phase error of each unit cell is calculated, as shown in Table [S6](#ts5).

**Table S6.** Phase Errors of the Alternative Design without PSO Algorithm [dB].

| Unit Cell Number | 1 | 2 | 3 | 4 | 5 | 6 | 7 | 8 | 9 | 10 | 11 | 12 | 13 | 14 | 15 |
| --- | --- | --- | --- | --- | --- | --- | --- | --- | --- | --- | --- | --- | --- | --- | --- |
| Phase Error [deg] | 0.5 | 1.9 | 4.3 | 6.4 | 7.9 | 10.5 | 18.3 | 27.8 | 37.6 | 47.7 | 56.7 | 48.0 | 40.0 | 33.2 | 28.7 |

Comparing the phase errors in Table 1 and Table [S6](#ts5), it is obvious that without using the PSO algorithm, phase errors have increased. Furthermore, it is worth mentioning that the phase error average of the design without the PSO algorithm is 24.85°, 6 times higher than the design with the PSO algorithm.

# An Alternative Design for Single Incident Angle

In order to illustrate the advantages of the proposed cloak over simple structures, a carpet cloak capable of rendering objects invisible for only one incident angle (zero degree) is designed, and its performance is compared with the proposed carpet cloak for other incident angles.

As shown in Fig. [S4](#fs2), CRR unit cells are used, which include a copper planar ring with a trace width of *w = 0.4 mm*, located on the substrate of Rogers RO4003C with a thickness of *t = 1.6 mm* and the relative permittivity of *ε_r_ = 3.5* and *tanδ = 0.0027*. The unit cell has a periodicity of *p = 6 mm*. These parameters remain constant, while parameter *a* is tuned to generate different reflection phases.

**Figure S4.** CRR Unit Cell.

By sweeping parameter *a*, the best unit cell designs are found to generate the required reflection phases according to equation (1) for incident angle of 0 degree. Table [S7](#ts6) represents the dimentions of the unit cells used in the design of simple cloak.

**Table S7.** Dimensions of unit cells used to make the simple cloak.

| Unit Cell Number | 1 | 2 | 3 | 4 | 5 | 6 | 7 | 8 | 9 | 10 | 11 | 12 | 13 | 14 | 15 |
| --- | --- | --- | --- | --- | --- | --- | --- | --- | --- | --- | --- | --- | --- | --- | --- |
| a [mm] | 5.80 | 1.60 | 3.70 | 4.25 | 4.40 | 4.55 | 4.60 | 4.70 | 4.80 | 4.90 | 5.00 | 5.25 | 5.70 | 1.60 | 3.70 |

After designing the carpet cloak, its performance for normal incidence is investigated by performing numerical simulation using Ansys HFSS software. Figure [S5](#fs3) illustrates simulation results, which indicate good performance of the designed cloak for normal incidence, as the near scattered electric fields and RCS pattern for the cloaked object are similar to the results achieved for a flat surface. Furthermore, its Reduced RCS is relatively low, as the Total Reduced RCS is 0.025. Therefore, the designed cloak is suitable for carpet cloaking for normal incidences. However, the simulation results for other incident angles, shown in Fig. [S6](#fs4), illustrates that the performance of the cloak is unacceptable for these angles. Figure [S6](#fs4) shows the normalized intensity of the scattered wave in the far-field region for incident angles of 0, 30, and 60 degrees, indicating the undesirable performance of the designed cloak for the oblique incidence, despite its good performance for the normal incidence.

**Figure S5.** Simulation results for the designed simple cloak under normal incidence. **a,c,e)** Near-field reflected electric field distribution for metallic ground plane, bare bump, and cloaked bump, respectively**. b,d,f)** The far-field normalized intensity of the scattered field (dB) for a flat ground plane, bare bump, and cloaked bump, respectively. **g)** Reduced RCS (dB) for both the bare bump (red) and the cloaked bump (blue).

**Figure S6.** The normalized intensity of the scattered wave (dB) for the cloaked bump under Gaussian wave incidence with incident angles of a) 0, b) 30, and c) 60 degrees.
